# Exploring the therapeutic relationship through the reflective practice of nurses in acute mental health units: A qualitative study

**DOI:** 10.1111/jocn.16223

**Published:** 2022-01-24

**Authors:** Diana Tolosa‐Merlos, Antonio R. Moreno‐Poyato, Francesca González‐Palau, Alonso Pérez‐Toribio, Georgina Casanova‐Garrigós, Pilar Delgado‐Hito, MiRTCIME.CAT Working Group

**Affiliations:** ^1^ Institut de Neuropsiquiatria i Addiccions Hospital del Mar Barcelona Spain; ^2^ Department of Public Health, Mental Health and Maternal and Child Health Nursing Nursing School Universitat de Barcelona L'Hospitalet de Llobregat Spain; ^3^ IMIM (Hospital del Mar Medical Research Institute) Barcelona Spain; ^4^ Hospital Santa Maria Salut/Gestió de Serveis Sanitaris Lleida Spain; ^5^ Unitat de Salut Mental de l'Hospitalet Gerència Territorial Metropolitana Sud Institut Català de la Salut L'Hospitalet de Llobregat Spain; ^6^ 16777 Department and Faculty of Nursing Universitat Rovira i Virgili Tortosa Spain; ^7^ Department of Fundamental Care and Medical‐Surgical Nursing Nursing School Universitat de Barcelona L'Hospitalet de Llobregat Spain; ^8^ GRIN‐IDIBELL (Nursing Research Group‐ Bellvitge Biomedical Research Institute) L'Hospitalet de Llobregat Spain

**Keywords:** action research, narrative, nurse–patient relationships, psychiatric nursing, reflective practice

## Abstract

**Aims and objectives:**

To explore the therapeutic relationship through the reflective practice of nurses in acute mental health units.

**Background:**

In mental health units, the therapeutic relationship is especially relevant for increasing the effectiveness of nursing interventions. Reflective practice is considered an essential aspect for improving nursing care.

**Design:**

Action and observation stages of a participatory action research project.

**Methods:**

Data were collected through reflective diaries designed for the guided description and reflection of practice interactions related to the therapeutic relationship and content analysis was applied. A total of 152 nurses from 18 acute mental health units participated. The COREQ guidelines were used.

**Results:**

The results were classified into three categories as follows: (i) Nursing attitude as a core of the therapeutic relationship. For the nurses, the attitudinal component was key in the therapeutic relationship. (ii) Nursing practices that are essential to the therapeutic relationship. Nurses identified practices such as creating a conducive environment, using an appropriate verbal approach, offering help and working together with the patient as essential for establishing a therapeutic relationship in practice. (iii) Contextual factors affecting the therapeutic relationship. The nurses considered the patient's condition, the care dynamics of the unit and its regulations, as well as the structure and environment of the unit, as contextual factors involved the establishment of an adequate therapeutic relationship in daily clinical practice.

**Conclusions:**

This study has provided knowledge of the importance and role of the nurses' attitude in the context of the nurse–patient therapeutic relationship based on the reflections of nurses in mental health units regarding their own practice.

**Relevance to clinical practice:**

These findings help nurses to increase awareness and develop improvement strategies based on their own knowledge and day‐to‐day difficulties. Moreover, managers can evaluate strategies that promote motivation and facilitate the involvement of nurses to improve the therapeutic relationship with patients.

**What does this paper contribute to the wider global clinical community?**

- An in‐depth analysis of nurses' reflections regarding the aspects that underlie the therapeutic relationship in their clinical practice enables the nurses themselves to become aware and to develop strategies for improvement based on their own knowledge.
- Understanding and confirming how the attitudinal component is a key element for nurses in the practice of the therapeutic relationship allows managers to evaluate strategies that promote motivation and facilitate the involvement of nurses to improve their practice with patients.
- The results point to the need for further studies aimed at identifying and implementing strategies that facilitate mental health nurses to incorporate and improve attitudinal skills related to establishing the nurse–patient therapeutic relationship in clinical practice.

## 1 INTRODUCTION

The nursing discipline is defined as a significant, therapeutic and interpersonal process that acts in conjunction with other human processes that make health possible for individuals (Peplau, 1988). The relationship established between nurse and patient is therapeutic, regardless of the setting in which care is provided (Stevenson & Taylor, 2020). However, in the mental health unit setting, the therapeutic relationship is especially relevant to increase the effectiveness of any nursing intervention (McAndrew et al., 2014). Reflective practice is considered an essential aspect of improving nursing care and generating knowledge (Vaughan, 2017). This paper aims to deepen the knowledge of the therapeutic relationship based on the reflections of nurses regarding their practice, in the context of current challenges within the mental health acute care setting.

### 1.1 Background

Based on Peplau's model of interpersonal relationships by (1988), which is the most widely held theory in the mental health nursing community, many authors have based their models on person‐centred mental health nursing (Barker & Buchanan‐Barker, 2010; O'Brien, 2001; Scanlon, 2006). All of them identify the therapeutic relationship as the foundation of nursing practice and the pillar upon which mental health nursing has been built (McAllister et al., 2019; Moreno‐Poyato et al., 2016). The proper establishment of the nurse–patient therapeutic relationship is especially relevant to increase the effectiveness of any nursing intervention in acute psychiatric units (McAndrew et al., 2014).

The therapeutic relationship could be defined as a human exchange (Peplau, 1988) that is based on effective communication that favours the possibility for a person to help another person to improve their health condition, with the objective that, through such communication, the person will be able to develop interpersonal and problem‐solving skills (Forchuk et al., 1998). To this end, concepts such as understanding, interest, availability, individuality, authenticity, warmth, respect and self‐knowledge are basic pillars for the nurse (Moreno‐Poyato et al., 2016). The literature points out that mental health nurses seem to be knowledgeable of the importance of the therapeutic relationship in inpatient units; however, the reality of clinical practice leads us to believe that theoretical knowledge is not enough to create a good bond with patients (Moreno‐Poyato et al., 2016). In addition, the literature points out that for nurses, the implementation of the therapeutic relationship in the current context of mental health units has suffered a strong impact related to neoliberal policies, with increased management and a risk‐centred approach (Kingston & Greenwood, 2020). Thus, today's environments are chaotic, and nurses are committed to therapeutic work, yet they struggle to balance it with the new demands of management (Kingston & Greenwood, 2020). In addition, barriers such as lack of time, communication problems (Harris & Panozzo, 2019a), the physical structures of the units, the ratios or the cultures of care are external factors that limit the therapeutic relationship (Tolosa‐Merlos et al., 2021). If nurses are unable to become aware of how they respond to time pressure, frustration or unclear care policies, there is a risk that these barriers will become entrenched, new ones will be created and the patient will perceive their actions as lacking care, presence or involvement (Harris & Panozzo, 2019b). Thus, although nurses recognise the importance of self‐awareness and knowing how to recognise how their actions can impact the therapeutic relationship and the care provided to patients, they are also aware of the need for self‐awareness (Thomson et al., 2019), institutions and, in general, care policies should encourage nurses to be aware of interpersonal influences, as well as the desirability of providing a safe and supportive clinical environment for these relationships (Stevenson & Taylor, 2020).

From the patients' point of view, in the complex environment of inpatient units, their interactions with staff are central components to their satisfaction regarding their experience with admission (Molin et al., 2021). When staff spend time, engage in daily activities, and recognise patients as individuals, patients seem to find it easier to be physically and emotionally closer to each other and to themselves (Eldal et al., 2019; Moreno‐Poyato et al., 2021). However, this therapeutic commitment is not always met in practice, and interventions to improve participation are few and far between and ineffective (McAllister et al., 2021).

Thanks to the therapeutic relationship, nurses are in a key position to lead the development of customised interventions (Molin et al., 2021). However, there is a significant gap in the literature regarding improving the quality of the therapeutic relationship in acute mental health units (Hartley et al., 2020). The nursing profession is characterised by its ability to reflect on practice to improve care and provide more person‐centred care, which is why there is a need to increase the use of evidence‐based practice (Vaughan, 2017). In fact, reflective practice allows practitioners to learn from their experiences (Bulman & Schutz, 2013; Schön, 1987). When nurses are given time to reflect through guided reflection questions they are able to gain valuable insight into practice (Bolg et al., 2020); therefore, reflective practice helps nurses integrate their emotional response and practical experience into a better understanding of the care they provide, incorporating knowledge and applying theory (Vaughan, 2017). Thus, although the nurse–patient therapeutic relationship has been extensively studied, no studies to date provide knowledge on the establishment of the therapeutic relationship and its implications based on the reflection on the nurses' own practice. Consequently, knowing the meaning of the therapeutic relationship together with the elements that facilitate and hinder its implementation in the complex practice of current acute mental health units can be a starting point for both nurses and managers to become aware of the needs and for the design of strategies for improvement, suited to the reality of clinical practice.

In this regard, the aim of this study was to explore the phenomenon of the therapeutic relationship through the reflective practice of nurses in acute mental health units.

## 2 METHODS

### 2.1 Design

This study is part of a multicentre mixed methods study involving 18 acute mental health units in Catalonia (Spain) (MiRTCIME.CAT). The principal aim of the project is to improve the nurse–patient therapeutic relationship through the implementation of evidence. The project was carried out following a sequential and transformational design. Quantitative methods were used based on a single‐group quasi‐experimental design with baseline and follow‐up measurements in phases I and III of the project. In the second phase, qualitative methodology was used. In its qualitative component, participatory action research (PAR) was proposed, framed within the constructivist paradigm and following the model by Kemmis and Mctaggart (2008). A two‐cycle process consisting of four stages each was designed to carry out the PAR. Specifically, this work corresponds to the action and observation stages of the first cycle. These stages are basic in the PAR process of change and make it possible to generate relevant knowledge regarding habitual practice (Cusack et al., 2018). In fact, it allows nurses to understand their practices as the product of particular circumstances and thus to identify the crucial aspects on which it may be possible to transform the practices they are carrying out (Kemmis & Mctaggart, 2008). The study is reported in line with the Consolidated criteria for reporting qualitative research guidelines (COREQ: Tong et al., 2007) (File S1).

### 2.2 Participants

All the acute mental health units that were part of the Catalan Mental Health Network (*n* = 21) were informed of the study. The principal investigator presented the research project and its objectives to the management of each centre through informative sessions. Finally, 18 units agreed to participate. A nurse from each unit joined the research team and this researcher was in charge of coordinating the study at their centre and recruiting the nurses from each unit. All nurses employed in the participating units (*n* = 235) were invited to participate in the study. The inclusion criteria for the participating nurses were belonging to the permanent or interim staff and being assigned to the acute unit at the time the intervention began. The following nurses were excluded from the study: nurses who were training to obtain ‘the official qualification of mental health nurse’, staff nurses who were scheduled to be on leave or maternity leave during the intervention. Thus, a convenience sample of 195 nurses agreed to participate in PAR, of which, ultimately 152 nurses completed the action and observation stages of the first part of this study.

### 2.3 Data collection

During a previous meeting among the entire research team, a guide was agreed upon so that the nurses could self‐observe their clinical practice in relation to the establishment of the therapeutic relationship. The research team sent the self‐observation guide by email to each nurse, along with a reflective diary in which the nurses were asked to record the self‐observation data (File S2). The diary was to include the description and reflection of three types of common interactions in their usual clinical practice: (a) a standard situation of welcoming a patient for admission, (b) an interaction in which there was a pre‐agitational state that required verbal de‐escalation and (c) an interaction whereby the patient is approached individually, promoted by the nurse and in the absence of any demand on behalf of the patient. The structure of the diary, together with the instructions for completion, pursued two purposes. First, to enable nurses to reflect on their starting assumptions, to understand their practice, to understand themselves and their patients, and, finally, to understand their profession (Price, 2017). Second, to monitor the process of change planned for the PAR, according to the proposals of Kemmis and Mctaggart (2008). In this sense, for each interaction, the nurses had to record the description of the situation, the type of verbal and nonverbal language they had used, their reflected intervention, their emotions during the interaction and, finally, a reflection on the influence of the environment on the interaction. Once the nurses had completed the diary, they sent it to the research team by e‐mail. The data were collected between April and June 2018.

### 2.4 Ethical considerations

This study was approved by the Research Ethics Committees of all the participating hospitals. The nurses participated on a voluntary basis, and all participants signed an informed consent form. Nurses did not receive any compensation or incentive for participating in the study. To maintain the confidentiality and anonymity of the data obtained, each nurse received an alphanumeric code that was incorporated into their diary. The diaries were sent to a generic e‐mail of the project that was only accessible to the principal investigator of the project, subsequently, the data were stored on a computer used exclusively for this study.

### 2.5 Data analysis

The content analysis method was used to analyse the data (Crowe et al., 2015). The diaries reached the first author and were coded to preserve the anonymity and confidentiality of the participants. Under their responsibility, the entire coding and categorisation process was carried out in a consensual manner by a collaborative team that formed the backbone of the process of developing a rigorous coding system (Merriam, 2016). In the first stage of analysis, the text was fragmented into descriptive codes assigned exclusively according to their semantic content. In a second stage, these initial codes were grouped into more analytical subcategories, which classified the codes according to the meaning of the linguistic units and their combinations. This led to a third hierarchical stage in which, considering the semantic analysis of the previous subcategories, the codes were ranked inductively. The first and second steps were taken iteratively until a more specific understanding of the subcategories was achieved. These steps were carried out primarily by the first author and discussed and reflected upon continuously and critically within the research team. Throughout the process, the QRS NVivo 12 program was used as computer support.

### 2.6 Rigour

Reflexivity was continuous throughout the process. Most of the researchers were experts in mental health, with training in qualitative methodology and experience in previous similar studies. As this was a multicentre study and a very large research team, neutrality was ensured as team members adopted an open attitude towards sharing, reasoning and discussing the findings as they emerged. In addition, the team became aware of its initial onto‐epistemological positioning, which was reflected in the design of the self‐observation guide for this stage of the process. As the research progressed, team members repeatedly contrasted the experiences identified in the participants' diaries with their own opinions. They asked follow‐up questions for the generation of new knowledge without guiding the participants' responses, so that this initial positioning could not influence the subsequent analysis. Similarly, the credibility and confirmability of the data should be emphasised, given the triangulation of the researchers in the analysis process and the constant auditing of the results by the participants in subsequent groups. In relation to the transferability of the results, in the case of this study, where participation is so high and from so many centres, it ensures that the results are valid for all units.

## 3 FINDINGS

The diaries of 152 nurses working at 18 centres were collected and analysed. The nurses ranged in age from 22 to 62 years, with a mean age of 33.6 years (SD = 9.4). Over 70% of the nurses were female. Their experience in mental health was a mean of 7.6 years (SD = 7.5). Almost a quarter of them had the official title of mental health nurse specialist and over 25% of the nurses had a doctoral or master's degree. All facility shifts were equally represented in the sample, although 40% of the nurses had rotating shifts or served on an as‐needed basis (Table 1).

**TABLE 1:** Participants' sociodemographic and professional characteristics (*n* = 152)

Variable	*n* (%)
Age, years	
20–29	68 (44.7%)
30–39	50 (32.9%)
40–49	23 (15.1%)
50–59	9 (5.9%)
60–69	2 (1.3%)
Gender	
Male	40 (26.3%)
Female	112 (73.7%)
MH nursing specialty	
Yes	36 (23.7%)
No	116 (76.3%)
Highest education	
Bachelor's degree	111 (73.0%)
PhD or Master's degree	41 (27.0%)
Work shift	
Morning	27 (17.8%)
Afternoon	36 (23.7%)
Night	28 (18.4%)
Rotating	61 (40.1%)
MH experience, years	
0–5	77 (50.7%)
6–10	31 (20.4%)
11–15	21 (13.8%)
16–20	12 (7.9%)
21–25	4 (2.6%)
26–30	1 (0.7%)
<30	3 (2%)

Note

Data are shown as absolute number (percentage).

Abbreviation: MH, mental health.

**FIGURE 1:**
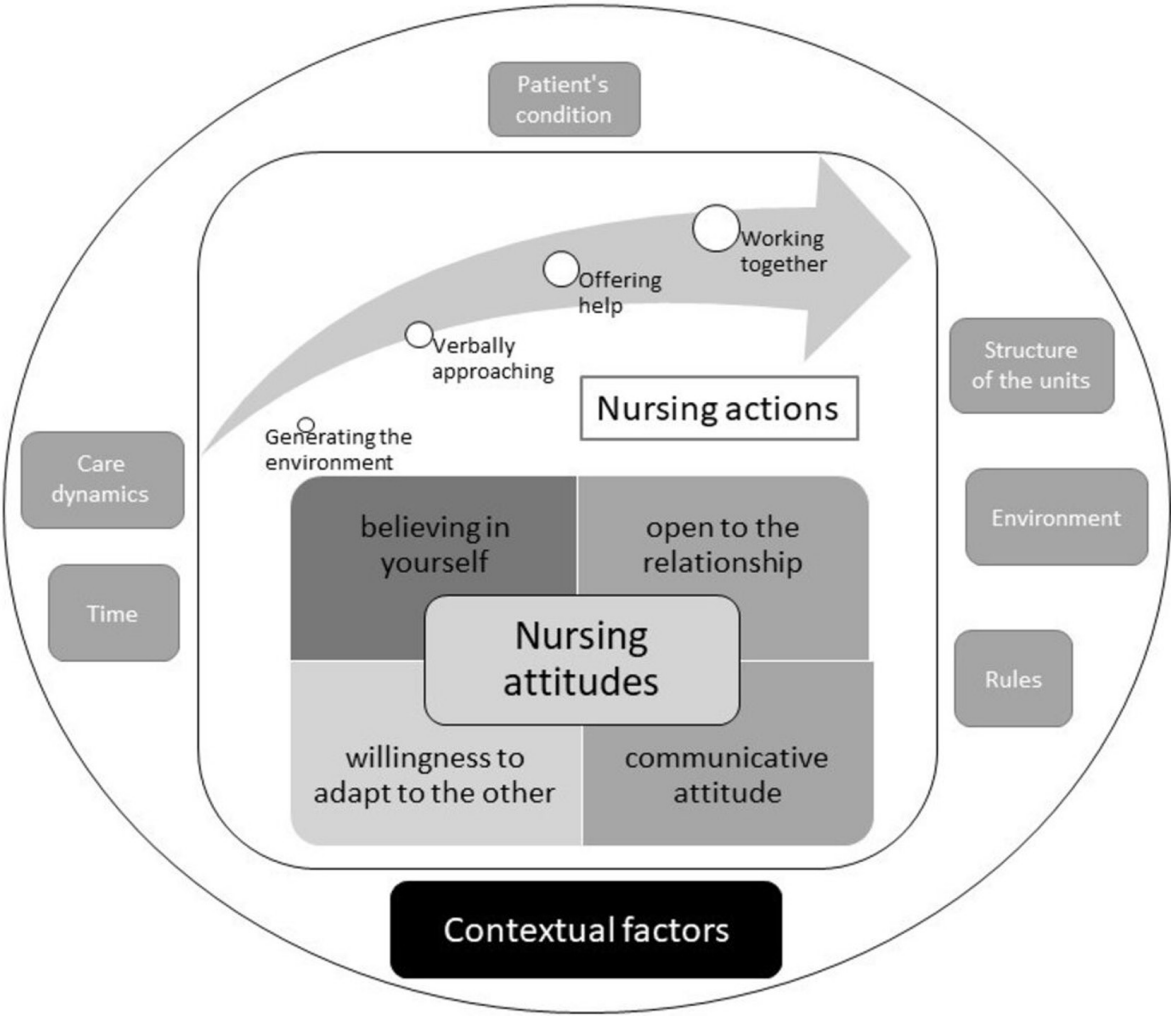
Nurses' reflections on the practice of the therapeutic relationship in acute mental health units

The nurses, by describing and reflecting on their interactions with patients, expressed what the therapeutic relationship was for them and how it was carried out in their usual clinical practice. In this sense, three main categories were identified that responded to how they gave meaning to what the therapeutic relationship represented in practice and what limitations they identified in it (Figure 1).

### 3.1 Nursing attitude as a core of the therapeutic relationship

After reflecting on their practice, the nurses stated that attitude was a key element in establishing a quality therapeutic relationship with patients in the units. In this regard, they identified different attitudinal components. In the first place, the nurses considered the attitude of openness to the relationship. This meant being open and available, offering time, letting the patient talk and being attentive to the person's needs.

Patients are confused when they are first admitted and need the staff to listen to them and spend time with them. I always try to use an empathetic approach and be honest from the very beginning. I think it is very important for the patient to know that they can count on me, I try to convey that I am available if they need me. (01DR101)

However, they also identified that, in order to maintain this attitude, they had to be aware of barriers such as the presence of prejudice, the unavailability of other team members, the belief that the therapeutic relationship is useless, or lying to the patient.

The first contact already gives me the feeling that there may be a personality background, a victimizing attitude, excessively correct at times, totally inadequate at others, in spite of which I stay on track and treat him with the utmost respect. (10DR101). Certain users only perform certain actions to push you to the limit. (13DR103)

Secondly, they referred to the communicative attitude as another basic element in the therapeutic relationship. In this case, the nurses considered that special attention should be paid to both their verbal and nonverbal language when interacting with patients. In this sense, they pointed out the need to establish a dialogue with the patient by means of clear and concrete messages, with an appropriate tone and without shouting, as well as showing interest in the conversation, listening attentively, without showing tiredness or boredom, and adapting their distance and physical contact to each situation.

I try to be aware of my gestures, I avoid being invasive, respecting the safety distance with the patient at all times. Regarding verbal language, I use neutral terms, a friendly and calm tone of voice. (04DR115). In a polite but firm manner, I explain to the patient his situation and the alternatives I can offer him instead of smoking. The language is clear and concise, responding directly to what he asks. Saying NO if necessary, as sometimes vague answers upset the patient even more. (04DR104)

In addition, they considered it extremely important that, as caregivers, they should adapt to the other person, that is individualise the care they provide in the context of the therapeutic relationship. This implies considering the patient's psychopathological and emotional state at any given moment, as well as the patient's age, language or culture. This often meant postponing interviews, adapting language, using sign language to communicate, agreeing on a special type of diet, or even relaxing the rules and letting the patient make a call outside the usual hours.

I try to be flexible and adapt things as much as I can to the patient and his or her characteristics. (03DR109). Sometimes the stigma in mental health appears from the self‐stigma and the treatment that the mental health professional gives to patients. Personality is lost by prioritizing the disorder, people talk about the schizophrenic, the depressive, the BPD… obviating the fact that there is a person behind it all, with a context and a manner of understanding and living their life. (05DR104)

Finally, the nurses emphasised the role of their own emotional experience of caregiving. This meant having self‐confidence, feeling they were able to help the patient and do their job well, feeling satisfied with their work and remaining calm, at ease, and relaxed with the patient during their interventions. Nurses also identified emotions that, conversely, had a negative effect on the therapeutic relationship, such as feeling fear, insecurity, tension, patient rejection, grief, helplessness and frustration when the interventions had not been resolved as expected.

To feel fulfilled in my daily work (18DR101). Calm and confident, well supported by the team. Satisfied to have successfully completed an admission. (16DR112). Then I felt helpless, as I could not find a way to reverse the situation. (12DR111)

### 3.2 Essential nursing actions for the therapeutic relationship

This category refers to the nurses' reflections on their actions in the context of the therapeutic relationship with patients. In their diaries, the nurses were describing and reflecting on different interventions and activities that were carried out in their usual practice and they detected certain actions that were common to all of them.

First, the nurses pointed out the importance of generating an appropriate environment to build a bond and facilitate the relationship with the patients. A calm, intimate, comfortable, unhurried environment without external stimuli or interruptions.

The room is quiet with the door closed and without any interaction from the environment…A pleasant and silent environment favors the therapeutic relationship between the professional and the patient. (04DR110)

In relation to the establishment of a good therapeutic bond, the nurses agreed that the welcome provided on admission was a fundamental intervention. This was viewed as one of the situations in which the therapeutic relationship took on a greater relevance, since this first contact was considered the key to the success of the subsequent relationship with the patient.

Without welcoming the patient when he or she enters the unit, a better quality of the patient/professional relationship cannot be achieved. (01DR113)

Secondly, the nurses felt that the verbal approach was also a relevant aspect of their practice in the context of the therapeutic relationship. For them, it was an essential step in order to be able to carry out any intervention, such as when welcoming a patient when they are admitted to the unit, the use of verbal de‐escalation techniques to ease the tension with very demanding and uncooperative patients or, on the contrary, to approach isolated patients who hardly interact with the environment, although the use of words is not always as effective as they would like it to be.

Verbal containment is one of the most relevant parts of our work. In a pre‐agitation situation, we may be able to transition a patient from pre‐agitation to calmness or from pre‐agitation to psychomotor agitation. (09DR108)

In this sense, the nurses described that the act of offering the patient their assistance was at the heart of the therapeutic relationship. They stated that this action was carried out in the context of being present, listening or through agreement with the patient by proposing alternatives to the demands and needs that they cannot meet.

As he speaks I give him my support with non‐verbal language. I take his hand and he hugs me. I offer my help. We agree that he will make an effort to eat some solid food at dinner and that I will give him a supplement (he has it prescribed if he needs it). (01DR101)

The nurses also acknowledged that interventions such as mechanical restraint were sometimes the only measure to reduce stimuli or were implemented because of patient aggressiveness, risk of escape or even medical indication. However, the nurses reflected that, although this intervention was performed relatively often, it could be seen as a failure and a deterioration in the therapeutic relationship.

(…) avoid as much as possible the adoption of measures that restrict the mobility of the people under our care, since we are aware that this produces a significant deterioration of the therapeutic relationship, adding to the patient's mistrust and suspicion (…) (07DR105)

Finally, the nurses pointed to therapeutic work as another fundamental aspect of the therapeutic relationship. This meant working with the patient on positive reinforcement and other aspects such as pharmacological adherence, identification of symptoms or awareness of the disease, explaining the objectives of admission and the importance of asking for help, respecting the patient's decisions and involving the person in their care and recovery.

The attitude is one of interest, I keep an eye on her so that she doesn't get distracted and can talk calmly. I ask her what she thinks we can do for her to explore her expectations with the admission. (07DR101)

### 3.3 Contextual factors affecting the therapeutic relationship

The nurses identified contextual factors that facilitated or, on the contrary, acted as barriers to the therapeutic relationship. Indeed, they described that the type of admission could already condition the therapeutic bond, with voluntary admission being a facilitator. The same is true of other factors such as knowing the patient from previous admissions, and whether the patient remembers having a good experience in those previous admissions. However, the nurses also considered elements that are intrinsic to the patient, such as language, culture or bad experiences of previous admissions, as factors limiting the establishment of the therapeutic relationship.

He is open to help and agrees to the admission (03DR110). I must admit that the fact that I know the user from previous admissions has helped the situation to unfold smoothly. (14DR106)

Similarly, the nurses identified barriers that hindered or prevented the establishment and maintenance of a good therapeutic relationship, related to both the environment and the physical structures of the units. In this sense, the structural barriers were related to the lack of adequate spaces to carry out interventions with patients with the intimacy that the nurses considered necessary. Other environmental factors were noted, such as environmental noise and tension, the unpredictability of some patients, the presence of the family or the multiple interruptions were elements that added to the difficulty of the therapeutic relationship.

That afternoon the environment allowed me to dedicate some time to the patient, since there were no emergencies, other admissions, or complicated situations in the unit that required nursing intervention, apart from the "scheduled" or "usual" activities such as the control of vital signs, medication, etc. (03DR105)

Finally, the nurses also expressed how the regulations and care dynamics of the units also conditioned the therapeutic relationship in daily clinical practice. Thus, unit regulations were recurrently brought up by the nurses as a major barrier, due to the numerous limitations and prohibitions.

I explain the rules of the unit: no cell phones, no smoking, no entering other rooms, no belts, no glass objects, etc. and the established schedules… (10DR104)

Nonetheless, the greatest source of difficulties was the care dynamics at the unit, ranging from lack of time, high workload, administrative tasks, staff rotations or the night shift.

Even so, there are barriers that hinder the therapeutic relationship. Sometimes, our language is influenced by the tension in the unit, the lack of time, excessive administrative tasks, etc.… (01DR101)

## 4 DISCUSSION

This study aimed to explore the phenomenon of the therapeutic relationship from the reflective practice of nurses in acute mental health units. The nurses highlighted that attitude was the core aspect of the therapeutic relationship after reflecting on their practice. Similarly, they also reflected on the actions that were customary in the habitual interventions carried out in the context of the therapeutic relationship, identifying the most common barriers encountered in practice. Finally, the nurses reflected on those aspects of the context of care that conditioned the therapeutic relationship in the clinical practice of acute mental health units.

These findings offer knowledge about relational competence, a competency of professional nursing that is highly relevant in mental health (D'Antonio et al., 2014). This competence is directly linked to participation in practice and incorporates not only knowledge and skills, but also attitudes and professionalism that involve applying evidence and learning to practice (Casey et al., 2017; Moreno‐Poyato, Casanova‐Garrigos, et al., 2021). Specifically, the attitudinal component highlighted in the results and its importance in the context of the nurse–patient therapeutic relationship has been described from a theoretical perspective by authors such as Peplau or Orlando (Forchuk, 1991), Travelbee (1971) and Watson (Turkel et al., 2018). Similarly, the empirical literature has collected multiple studies that study the importance of nurses' attitudes towards more general aspects of mental health, such as stigma (Young & Calloway, 2021), recovery (Gyamfi et al., 2020), coercion (Doedens et al., 2020; Laukkanen et al., 2019) or severe mental disorder (Economou et al., 2019). However, there is hardly any empirical evidence that explicitly shows the relevance and identifies the specific attitudinal skills of nurses in the context of the practice of the therapeutic relationship. Thus, it is likely that the fact that the nurses were able to reflect on their practice made them more aware of the importance of attitude in the context of the therapeutic relationship (Harris & Panozzo, 2019a), as they were able to respond to the real challenge of establishing an adequate therapeutic relationship in their day‐to‐day work in the acute mental health units (Choperena et al., 2019). Moreover, the attitudinal capacity identified by the nurses encompassed aspects already empirically recognised in the context of the therapeutic relationship, such as availability, communication and individualisation (Delaney & Johnson, 2014; Harris & Panozzo, 2019b; McAllister et al., 2019; Moreno‐Poyato et al., 2016). However, the nurses also highlighted other aspects that have been less empirically studied, such as the importance of self‐confidence and self‐assurance, both in a positive way in order to be able to establish an appropriate therapeutic relationship, (Roche et al., 2011; Van Sant and Patterson, 2013) as well as negatively, in the form of limitation (O'Connor & Glover, 2017; Van Sant and Patterson, ). These results confirm the relevance of Peplau and Orlando's theoretical approaches and the use of the nurse's awareness as a fundamental part of the nursing relationship (Forchuk, 1991; Thomson et al., 2019).

The results indicate that by reflecting on their practice, the nurses were able to identify those skills (practices) that are essential for the development of the therapeutic relationship and which were transversal to any intervention. The nurses emphasised the importance of generating an adequate environment for the relationship, considering the environment not only as an element of context typical of many acute care units, but also as an element that is essential for the development of the therapeutic relationship (Kingston & Greenwood, 2020), also considering that it was their responsibility to be able to build the space where the relationship could take place (McAllister et al., 2021; Raphael et al., 2021). As in other studies, nurses also identified skills such as verbal engagement, offering help or working with the patient as basic practices for the development of effective interventions in the context of the relationship with their patients (Harris & Panozzo, 2019a; McAllister et al., 2019; Molin et al., 2018). Furthermore, in relation to specific interventions, reflection on practice allowed nurses to identify and become aware of nursing admission assessment and mechanical restraint as two common interventions in mental health units that were particularly influential in the therapeutic relationship with the patients. In this sense, for the nurses, welcoming the patient on admission was considered an essential intervention determining a large part of the success in building the therapeutic relationship with the patients (Forchuk et al., 1998; Peplau, 1997). However, the use of mechanical restraint compromised the therapeutic relationship and the patient's trust (Kinner et al., 2017), although they understood that, even if this measure was undesirable, at times it was necessary (Doedens et al., 2020).

In addition, the nurses reflected on the contextual factors that directly affected the therapeutic relationship with the patients. In this sense, the nurses paid attention to patient aspects such as voluntariness or involuntariness regarding admission (Moreno‐Poyato, El Abidi, et al., 2021) or being previously acquainted with each other from previous admissions and the experience of the relationship (Van Sant and Patterson, 2013). The nurses also emphasised the role of the environmental and structural conditions of the units (Staniszewska et al., 2019), as well as the regulations and the dynamics of care that were automatically generated in the intense day‐to‐day routine of the units (Adler, 2020; Kingston & Greenwood, 2020).

### 4.1 Strengths and limitations

This study has several strengths and limitations. First, it should be noted that this project faced major challenges from a methodological point of view as well as during its execution. Initially, a research group had to be formed with representation of the institutions to assess the feasibility of the project. Next, a balanced team of researchers, consisting of methodologists and clinicians had to be assembled to ensure that the different stages of the research project could be completed. The team had to be formed in several initial working sessions and, subsequently, there was a process of constant mentoring by the principal investigator to the rest of the team. In addition, a considerable volume of data had to be managed. For management and storage, a secure on‐line space was created, guarded and accessed only by the principal investigator of the project. All data were collected electronically to facilitate the circuit. In relation to the analysis, a team was set up under the responsibility of a researcher. This team had to work in a collaborative and consensual manner. Regarding more specific limitations, it should be mentioned that the nurses' reflections in the diaries could be subject to the Hawthorne effect and their responses may have been biased by social desirability. In this sense, the research team insisted on the importance of honesty in the nurses' responses and on the team's handling of the confidentiality of the data. Secondly, another limitation inherent to the use of diaries is related to memory bias and the stress associated with reflective practice. In relation to this, the team recommended specific instructions, both verbally and through the guide provided to the nurses, to prevent this from occurring. Furthermore, the representativeness of the participating nurses and the number of diaries obtained should be highlighted as strengths of the study. These facts enable the findings of this study to be transferred to similar contexts.

## 5 CONCLUSIONS

The present study contributes to the understanding of the phenomenon of the therapeutic nurse–patient relationship by reflecting on the actual practice of nurses in acute mental health units. The attitudinal component is at the heart of the therapeutic relationship, and, in this sense, it is fundamental for nurses to believe in themselves and their attitude to communicate, adapt and open up to the relationship with the patient. In addition, there are actions that are essential for nurses to establish a TR in practice such as creating a conducive environment, using an appropriate verbal approach, offering help and working together with the patient. Finally, nurses should consider the patient's conditions, the dynamics of care and regulations of the unit, as well as the structure and environment of the unit, as contextual factors to be able to establish an adequate TR with patients in daily clinical practice.

## 6 RELEVANCE TO CLINICAL PRACTICE

These findings have important implications. The study findings demonstrate that participatory methods stimulate nurses' reflection, motivation and critical thinking. By learning from the reflection of the nurses themselves about the aspects that underlie the therapeutic relationship in their clinical practice, this enables the nurses themselves to become aware and to develop strategies for improvement based on their own knowledge. Moreover, the individual reflection involved in these first stages of a participatory process provides the nurses with an intrinsic knowledge of how they approach the therapeutic relationship and shows that the attitudinal component is basic for them. In this sense, understanding and confirming how the attitudinal component is a key element for nurses in the practice of the therapeutic relationship allows managers to evaluate strategies that promote motivation and facilitate the involvement of nurses in improving their practice with patients. Moreover, these results point to the need to conduct mixed or qualitative studies aimed at exploring the aspects that facilitate the motivation, empowerment and attitudinal training of nurses in greater depth, rather than studies that only seek improvements in the theoretical knowledge of the therapeutic relationship.

## CONFLICT OF INTEREST

No conflict of interest has been declared by the authors.

## AUTHOR CONTRIBUTIONS

Study design: ARMP and PDH; Data collection: APT, FGP and GCG; Data analysis team: DTM; Final report draft: DTM, ARMP and PDH; Supervision the process of data collection and analysis and provide support and feedback during all study phases: ARMP; Contribution of the manuscript, and read and approved the final manuscript: All authors.

## Supporting information

File S1

File S2

## Data Availability

The data that support the findings of this study are available from the corresponding author upon reasonable request.
