# Supplementary material for: Exploring the therapeutic relationship through the reflective practice of nurses in acute mental health units: A qualitative study
Source: J Clin Nurs. 2022 Jan 24;32(1-2):253–63. doi: 10.1111/jocn.16223 (PMC10078778; doi:10.1111/jocn.16223)
Supplement: Supplementary file 1 — File S1 [file JOCN-32-253-s002.docx]

This journal is meant to provide a **description and reflection**of aspects of the therapeutic relationship of interactions made in clinical practice by the nurses participating in this study.

You are asked to describe each interaction in such a way that anyone who has not witnessed the scene is able to visualize it.

In this case, the reflective journal constitutes one of the main research tools of the project. This exercise **is not** meant to judge your health care practice, rather its aim is to **know**how the **therapeutic relationship**takes place within acute units.

In this sense, you are asked to answer the following open questions, **honestly**. Before writing them down, you must give yourself a **few moments of reflection**, it is not necessary to do so quickly, you must allow yourself some time, take notes of the most important things so that you can later remember and write them down. It is advisable to self-observe several similar interactions to practice the self-observation exercise and finally, describe the one that interests you the most and respond in relation to it. You can write whatever you consider appropriate, there is no word limit.

We remind you that this document is **anonymous.**For this reason, we will send you your personal code again and once you have the finished diary, you can send it to us by replying to the email you received and indicating your participant code. In this way, your email will be received directly by the research team and no one else will see these responses.

The **interactions**that have been planned for this self-observation are as follows:

1. Standard nursing admission assessment.
2. Standard situation in which there is a pre- agitation state that requires verbal de-escalation.
3. Situation of individual approach promoted by you, with or without any technique involved and without demand from the patient.

In each of these interactions you should do the exercise of self-observing your clinical practice and answer the following questions.

| **SELF-OBSERVATION OF CLINICAL PRACTICE Interaction 1** |
| --- |
| **Type of interaction:**Nursing admission assessment**Date:** |
| **Briefly describe the situation:** |
| **My language and speech during the interaction:**  What is the verbal and non-verbal language that I am using? |
| **My performance during the interaction:**  What concrete steps do I take and how do I go about them? Why do I do it like this? |
| **Influence of the environment during the interaction:**  What happens in the environment while the interaction is taking place and how does it affect me?  How does the therapeutic relationship arise within my institution? Has this positioning influenced this interaction? |
| **My feelings during the interaction:**  What emotions has the development of the interaction generated in me throughout the process? How did I feel? |

| **SELF-OBSERVATION OF CLINICAL PRACTICE                               Interaction 2** |
| --- |
| **Type of interaction:**Pre- agitational state that requires verbal de-escalation**Date:** |
| **Briefly describe the situation:** |
| **My language and speech during the interaction:**  What is the verbal and non-verbal language that I am using? |
| **My performance during the interaction:**  What concrete steps do I take and how do I go about them? Why do I do it like this? |
| **Influence of the environment during the interaction:**  What happens in the environment while the interaction is taking place and how does it affect me?  How does the therapeutic relationship arise within my institution? Has this positioning influenced this interaction? |
| **My feelings during the interaction:**  What emotions has the development of the interaction generated in me throughout the process? How did I feel? |

| **SELF-OBSERVATION OF CLINICAL PRACTICE Interaction 3** |
| --- |
| **Type of interaction:**individual approach promoted by you, with or without technique involved and without demand from the patient  **Date:** |
| **Briefly describe the situation:** |
| **My language and speech during the interaction:**  What is the verbal and non-verbal language that I am using? |
| **My performance during the interaction:**  What concrete steps do I take and how do I go about them? Why do I do it like this? |
| **Influence of the environment during the interaction:**  What happens in the environment while the interaction is taking place and how does it affect me?  How does the therapeutic relationship arise within my institution? Has this positioning influenced this interaction? |
| **My feelings during the interaction:**  What emotions has the development of the interaction generated in me throughout the process? How did I feel? |
